# Short-term side effects of BNT162b2 vaccine in primary care settings in Qatar: a retrospective study

**DOI:** 10.3389/fpubh.2024.1384327

**Published:** 2024-04-10

**Authors:** Sami Abdeen, Muna Abed Alah, Manal Al-Zaidan, Mohamed Izham Mohamed Ibrahim, Jazeel Abdulmajeed, Asma Ali Al-Nuaimi, Mohamed Ghaith Al-Kuwari

**Affiliations:** ^1^Community Medicine Department, Hamad Medical Corporation (HMC), Doha, Qatar; ^2^Department of Pharmacy and Therapeutics Supply, Primary Health Care Corporation, Doha, Qatar; ^3^College of Pharmacy, QU Health, Qatar University, Doha, Qatar; ^4^Strategy and Health Intelligence Department, Primary Health Care Corporation, Doha, Qatar; ^5^Collège of Medicine, Qatar University, Doha, Qatar

**Keywords:** COVID-19, BNT162b2 vaccine, side effects, primary care, Qatar

## Abstract

**Background:**

Despite the established effectiveness of the BNT162b2 Vaccine, the novel technology demands careful safety monitoring. While global studies have explored its safety, local data remains limited and exhibits some variability. This study investigated short-term side effects among BNT162b2 vaccinated individuals in Qatar.

**Methods:**

A retrospective analysis was conducted using data extracted from the electronic health records of individuals aged 18 or older across 8 primary health centers who received either the first or second dose of the BNT162b2 vaccine during the period from December 23, 2020, to April 24, 2021. The proportions of individuals experiencing short-term side effects after each dose were calculated. Logistic regression and log binomial regression analyses were used to explore associations with the side effects.

**Results:**

Among 7,764 participants, 5,489 received the first dose and 2,275 the second, with similar demographics between the groups. After the first dose, 5.5% reported at least one local side effect, compared to 3.9% after the second, with a 1.4 times higher incidence after the first dose (RR 1.4, 95% CI 1.14–1.75) compared to the second. Systemic side effects after the second dose were 2.6 times more common than after the first (RR 2.6, 95% CI 2.15–3.14). Gender, nationality, history of prior COVID-19 infection, and obesity were significantly associated with side effects after the first dose, while age, gender, and nationality, were significant factors after the second dose.

**Conclusion:**

The rates of side effects following the BNT162b2 vaccine in Qatar were relatively low, with age, gender, nationality, previous infection, and obesity identified as significant predictors. These results emphasize the need for tailored vaccination strategies and contributes valuable insights for evidence-based decision-making in ongoing and future vaccination campaigns.

## 1 Introduction

The emergence of SARS-CoV-2 in late 2019 sparked an unprecedented global health crisis, leading to widespread morbidity and mortality. Numerous measures, such as mask-wearing, quarantine, and social distancing, have significantly contributed to the containment of SARS-CoV-2 infection (1). No specific drug is identified for the prevention or treatment of COVID-19, making vaccination the most cost-effective strategy to mitigate the transmission of SARS-CoV-2 (2). In response, the scientific and medical communities mobilized to develop effective vaccines. The Pfizer-BioNTech (BNT162b2) COVID-19 vaccine was one of the first mRNA-based vaccines to receive emergency use authorization, marking a significant milestone in pandemic control efforts (3). This vaccine uses mRNA technology and lipid nanoparticle (LNP) delivery systems. Its mechanism of action involves the introduction of a small piece of the virus’s genetic material into the body, that encodes the production of the SARS-CoV-2 spike (S) protein which is the primary target for neutralizing antibodies generated from natural infection (4, 5). This, in turn, stimulates an immune response without exposing individuals to the live virus. The initial clinical trials demonstrated exceptional efficacy in preventing COVID-19 infection, and subsequent real-world data reinforced its effectiveness (6, 7). However, like all medical interventions, vaccines can have side effects, and understanding these is essential for a holistic evaluation of their benefits and risks. Given the substantial morbidity and mortality rates associated with COVID-19 infection, the scientific and public health communities were eager to expedite the distribution of a safe and effective vaccine to the population. Consequently, pandemic vaccines were rapidly deployed in large quantities upon their introduction (8).

Throughout the global vaccination rollout, numerous reports and research studies of vaccine side effects have surfaced, varying from mild symptoms such as pain at the injection site and fever to severe but rare events like anaphylaxis or myocarditis (9–12). The monitoring and assessment of these side effects are vital to ensure the vaccine’s safety, assess potential risk factors, and make informed recommendations regarding vaccine administration. Numerous studies have compared the safety profiles of the BNT162b2 vaccine with other COVID-19 vaccines. One cohort study suggested minimal differences in adverse event risks within 14 days of the first BNT162b2 dose compared to mRNA-1273 (13). Other studies indicated that BNT162b2 had lower rates of side effects than mRNA-1273, especially at short-term (14, 15). However, some studies showed that BNT162b2 had higher rates of side effects than other vaccine types such as Sputnik V, Sinovac and ChAdOx1-S/nCoV-19 (14). Additionally, the mRNA vaccines were found to be associated with a higher prevalence of local side effects, while the viral vector-based vaccine was linked to a higher prevalence of systemic side effects (16, 17).

Vaccine hesitancy and refusal pose significant challenges to the vaccination process during pandemics (18, 19). Acceptance rates of COVID-19 vaccines varied globally due to factors such as vaccine availability, mandatory vaccination policies, perceived effectiveness and cost, and experience of adverse events (19, 20). Vaccine side effects play a major role in its acceptance. Additionally, the expedited approval and the new technologies used for COVID-19 vaccines, especially mRNA vaccines, compared to traditional processes had also influenced hesitancy (21, 22). Furthermore, studies have shown that vaccine hesitancy differs depending on the type of vaccine, with varying rates of hesitancy observed for different vaccines (23). For example, it was found that initial vaccination with mRNA-1273 was associated with greater hesitancy toward booster doses compared to BNT162b2 (24). These differences are primarily attributed to variations in safety profiles and side effects between the vaccines (25).

In Qatar, BNT162b2 vaccine was the cornerstone of the COVID-19 vaccination campaign, as it was the first vaccine to be approved for emergency use by the Department of Pharmacy and Pharmaceutical Control in the Ministry of Public Health from December 2020 onwards (26). The vaccine was administered in two doses with a 21-day interval to ensure optimal protection during that period. After January 2022, other vaccines, like the Moderna (mRNA-1273) and the Oxford–AstraZeneca (ChAdOx1-S/nCoV-19) were granted emergency use authorization in Qatar (27). This study aimed to evaluate individuals’ short-term side effects after receiving the BNT162b2 vaccine in Qatar. In some parts of the world, including Qatar, very few studies have been conducted on the side effects of COVID-19 vaccines, leaving a significant gap in our understanding of their safety in specific populations. This absence of local data can seriously affect public health decision-making, as the response to vaccine-related concerns may be less informed. Therefore, conducting a study on COVID-19 vaccine side effects in Qatar is crucial. Furthermore, this study will serve as a valuable foundation for addressing potential side effects in future pandemics and developing vaccines that are even safer and more effective for the global population.

## 2 Materials and methods

### 2.1 Study design, setting, and the target population

This study is a retrospective analysis of the data gathered from the Electronic Health Records (EHR) of Primary Health Care Corporation in Qatar for population aged 18 and older. In response to the unprecedented COVID-19 pandemic and the urgent need for vaccination, particularly with emergency use approval, the Ministry of Public Health in Qatar established a rigorous monitoring system to assess the potential side effects associated with administering BNT162b2 vaccine. This monitoring effort was of paramount importance and entailed the systematic collection of data recorded in patients’ electronic health records using a designated tool known as the “COVID-19 Post Vaccine Assessment Form.” This form featured a series of Yes/No questions relating to specific symptoms, encompassing both local and systemic side effects.

The vaccine side effect monitoring process took place using multiple pathways:

- Direct contact with vaccinated patients: At each vaccination center, a healthcare team is assigned to initiate contact with all vaccinated patients from the respective center between the third and fifth day following the first and second doses of the vaccine, through telephone calls. To commence this process, the team is provided daily with a contact information list for all individuals who received the vaccine at the center. During these calls, the team collected data, specifically inquiring about side effects, using the “COVID-19 Post Vaccine Assessment Form.” This data was documented immediately in the patient’s electronic health record. The symptoms included injection site pain, swelling, redness, localized swollen lymph nodes, fatigue, fever, headache, myalgia, dizziness, chills, nausea, vomiting, abdominal pain, diarrhea, arthralgia, and others.
- Self-reporting by patients using a dedicated hotline: Patients had the option to proactively report any side effect by calling a designated hotline and a healthcare staff would electronically document the reported side effects using the same “COVID-19 Post Vaccine Assessment Form” in the electronic health record system.

In this study, part of a larger project that aims to investigate the epidemiology and characteristics of side effects associated with COVID-19 vaccines in Qatar, we only selected the records of participants who were actively contacted by healthcare teams (first pathway). Accordingly, this study did not include side effects data documented through the second pathway (self-reporting by a hotline). This enabled us to calculate the rates of vaccine side effects accurately. Additionally, we selectively included data only from 8 primary health care centers. These were the only centers that provided us with daily, complete, and comprehensive telephone-calls lists which allowed us to precisely identify individuals who were contacted, those who responded, those who did not answer, and those who refused. We included participants who received the first dose of the COVID-19 vaccine in Qatar, starting from December 23, 2020, which marked the launch of the COVID-19 vaccination campaign in Qatar, and continuing until March 16, 2021, which marked the end of data collection for the first dose. And those who received the second dose in Qatar, commencing from January 12 (21 days after the first dose) and extending through April 24.

### 2.2 Data collection process and study variables

With the assistance of the Business Health Intelligence unit at Primary Health Care Corporation, we retrospectively extracted the data of individuals included in the study. Such data included the sociodemographic characteristics encompassed age, sex, and nationality and health-related data such as the date of any previously confirmed COVID-19 infection (if applicable), which we then used to compute the duration between the COVID-19 infection and the first vaccine dose and classified it as either less or more than 6 months. Regarding the presence of chronic diseases variable, it was categorized as No (having no chronic disease), or yes (having one or more chronic diseases). Chronic diseases were ascertained and classified based on the ICD-10 codes for the following chronic conditions (diabetes mellitus, hypertension, cardiovascular disease, respiratory diseases like asthma or COPD, cerebrovascular disease, cancer, kidney disorders, immune disorders, and liver disorders) as recorded in the electronic health record encounters of each individual in Cerner®. All encounters were analyzed for each individual to determine chronic disease classification. Individuals were classified based on at least one encounter with a specific chronic disease diagnosis. Individuals with chronic diseases who did not seek care in the public health care system or exclusively used private facilities were categorized as having no chronic disease due to the absence of recorded encounters. Moreover, access to complete medical records was limited, precluding an assessment of treatment variations, medications, or the duration of participants’ interaction with the medical system. Furthermore, data on height and weight were obtained to calculate BMI, classifying participants as obese or not obese, with a BMI of 29.9 as the cutoff point. Lastly, each individual’s vaccination side effects documented in “COVID-19 Post Vaccine Assessment Form” were also retrieved.

### 2.3 Outcomes

Our primary outcome was the proportion of vaccinated individuals experiencing side effects during 3–5 days after the first and second vaccine doses and the probability of having a specific adverse effect.

### 2.4 Ethical considerations

Ethical approval was obtained from the Primary Health Care Corporation research committee with protocol ID (PHCC/DCR/2022/04/024).

### 2.5 Statistical analysis

We used IBM SPSS Statistics for Windows, Version 26.0. Armonk, NY: IBM Corp to analyze the data. We summarized categorical data by providing frequencies and percentages, while numerical data were summarized using the mean and standard deviation. The Chi-square test was utilized to assess differences in the proportions of individuals who experienced each of the side effects after vaccination, comparing the first and second vaccine doses, and to determine the differences in the proportions of individuals who experienced localized, systemic, and both (systemic and localized) side effects across various levels of independent variables. Unadjusted odds ratios (ORs) and relative risks (RRs) were calculated to measure the strength of the association. In the case of multivariable analysis, multiple logistic regression and multiple log-binomial regression were utilized to evaluate predictors of side effects, following the necessary assumptions. The associations between predictors and outcomes were presented using adjusted odds ratios (ORs) and 95% confidence intervals (95% CIs) and relative risk (RR) with 95% confidence intervals (95% CIs). Statistical significance was considered at *p* < 0.05.

## 3 Results

### 3.1 Sociodemographic characteristics and background information

As shown in Table 1, for the first dose, the age distribution showed a concentration in the older age groups, with 2,093 participants (38.1%) aged between 60 and 74 years. The gender distribution was skewed toward males, with 3,919 participants (71.4%) being males. Most of the participants were expatriates, accounting for 3,403 individuals (62%). Regarding health history, a vast majority of 5,098 participants (92.9%) had no prior history of COVID-19 infection. History of chronic disease/s was reported in 3724 participants (67.8%), and obesity was noted in 782 individuals (14.2%). For the second dose, the age distribution was similar, with 999 participants (43.9%) in the 60–74 age bracket. The gender distribution remained predominantly male, with 71% being males. The expatriate participants continued to form the majority. A higher percentage of participants, 2,147 (94.4%), had no history of COVID-19 infection before the second dose. Chronic diseases were present in three-quarters of participants, and obesity was reported in 318 individuals (14%).

**Table 1:** Sociodemographic characteristics of included participants and background information.

Characteristics		First dose	Second dose
		M ± SD No (%)	M ± SD No (%)
Age		55 ± 16.2	59.6 ± 14.4
Age	less than 30	423 (7.7)	44 (1.9)
	30–44	1,162 (21.2)	385 (16.9)
	45–59	1,329 (24.2)	549 (24.1)
	60–74	2093 (38.1)	999 (43.9)
	75 or more	482 (8.8)	298 (13.1)
Sex	Female	1,570 (28.6)	660 (29)
	Male	3,919 (71.4)	1,615 (71)
Nationality	Local	2086 (38)	699 (30.7)
	Expatriate*	3,403 (62)	1,576 (69.3)
History of COVID-19 before vaccination	No	5,098 (92.9)	2,147 (94.4)
	Yes	391 (7.1)	128 (5.6)
Duration between COVID-19 infection and vaccination^‡^	6 months or less	112 (28.6)	26 (20.3)
	more than 6 months	279 (71.4)	102 (79.7)
History of chronic disease/s	No	1765 (32.2)	563 (24.7)
	Yes^†^	3,724 (67.8)	1712 (75.3)
Obesity (BMI 30 or more)	No	4,707 (85.8)	1957 (86)
	Yes	782 (14.2)	318 (14)

M, Mean; SD, Standard deviation.

^*^More than 150 different nationalities were reported.

^†^Most commonly reported chronic diseases were Diabetes, Hypertension, and Dyslipidemia.

^‡^The denominator is the number of participants who have history of confirmed COVID-19 infection prior to the administration of the first dose of the vaccine (*n* = 391 for the first dose group, *n* = 128 for the second dose group).

### 3.2 Distribution of side effects post BNT162b2 vaccination

**Figure 1:**
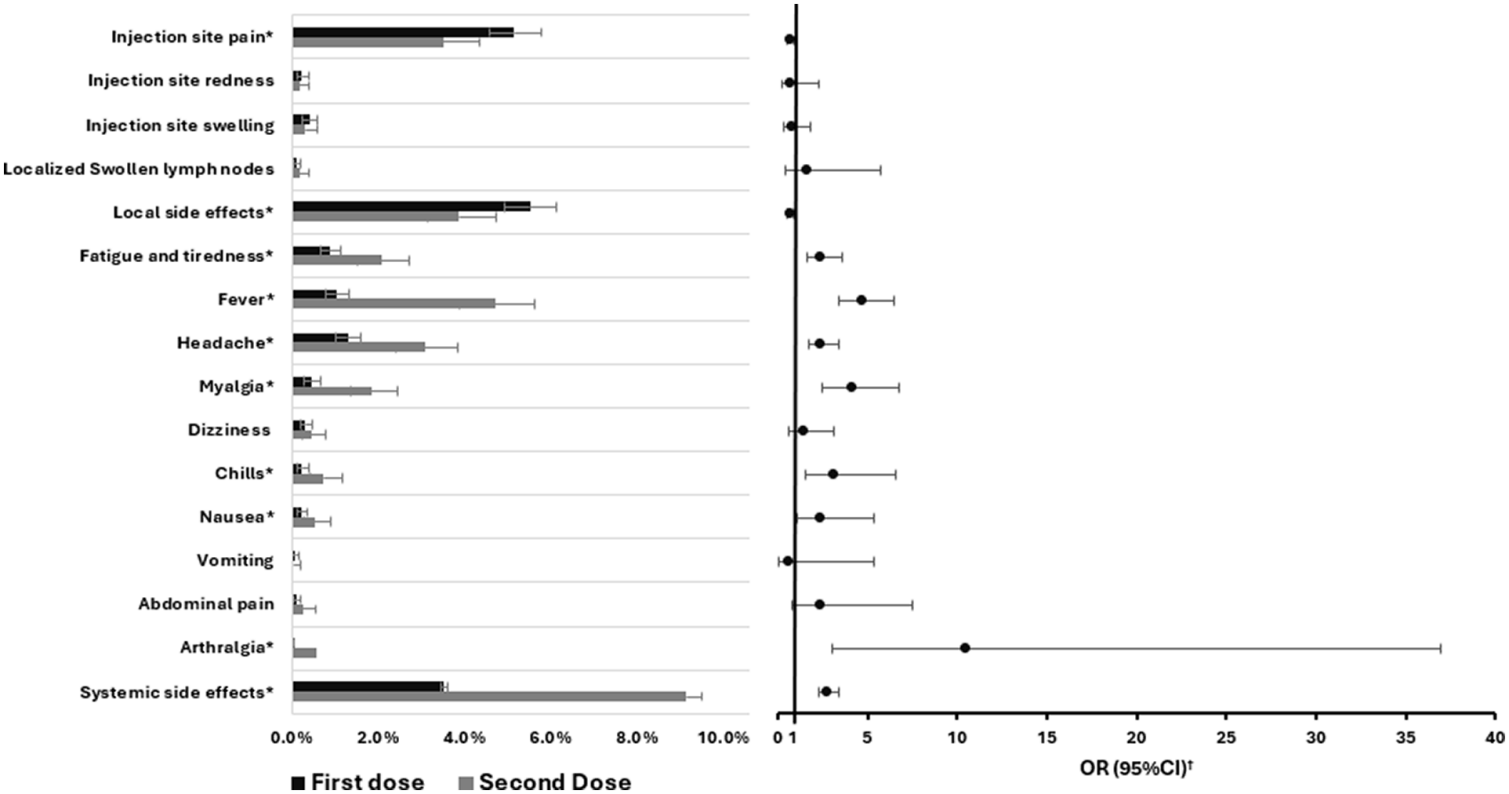
Comparison of the proportions of different side effects experienced by participants between the first and second BNT162b2 vaccine doses including error bars showing 95%CI and the odds ratios. OR, Odds ratio; CI, Confidence interval. *p-value <0.05. ^†^Second dose vs. the first dose.

In this study we conducted a thorough assessment of the side effects experienced by participants following the administration of both doses of the COVID-19 vaccine BNT162b2 (Figure 1; Supplementary Table 1). Injection site pain was the most commonly reported symptom, with 283 participants (5.2%) after the first dose and 80 participants (3.5%) after the second dose. This was followed by injection site swelling, which was observed in 22 participants (0.4%) post the first dose and 7 participants (0.3%) after the second dose. Overall, 303 participants (5.5%) reported at least one local side effect after the first dose, while 88 participants (3.9%) did so after the second. The incidence of local side effects following the first dose was about 1.4 times more compared to the second dose (RR 1.4, 95% CI 1.14–1.75). Turning to systemic side effects, after the first dose, they were reported by 193 participants, making up 3.5% of the participants. The most prevalent systemic symptom was headache, with 71 participants (1.3%) indicating its occurrence. Regarding the second dose, systemic effects were more pronounced, with 208 participants (9.1%) reporting them. Furthermore, fever emerged as the most common systemic side effect, experienced by 107 participants (4.7%). The incidence of systemic side effects following the second dose was about 2.6 times more compared to the first dose (RR 2.6, 95% CI 2.15–3.14) as shown in Supplementary Table 1. Furthermore, none of the included participants experienced anaphylaxis reaction following either the first or second dose.

### 3.3 Determinants of developing side effects post BNT162b2 vaccination

Following the administration of the first dose of the vaccine, we observed pronounced susceptibility among females and expatriates to experience side effects compared to their counterparts (males and Qataris) with *p* < 0.001. A notable finding was the heightened propensity for individuals previously diagnosed with COVID-19 to manifest side effects (*p* = 0.002). Moreover, a significant association was identified between obesity (BMI ≥ 30) and an increased likelihood of adverse outcomes (*p* = 0.005) as shown in Table 2.

**Table 2:** Determinants of developing one or more side effects following the first and second doses of BNT162b2 vaccine.

Characteristics		Any side effect					
		First dose			Second dose		
		Unadjusted OR (95%CI)	Unadjusted RR (95%CI)	*p*-value*	Unadjusted OR (95%CI)	Unadjusted RR (95%CI)	*p*-value*
Age	less than 30	0.64 (0.37–1.08)	0.66 (0.4–1.08)	**0.047**	1.14 (0.38–3.46)	1.13 (0.41–3.1)	**<0.001**
	30–44	1.18 (0.81–1.72)	1.16 (0.82–1.64)		2.41 (1.47–3.94)	2.16 (1.39–3.36)	
	45–59	1.03 (0.7–1.5)	1.02 (0.73–1.45)		2.61 (1.63–4.17)	2.31 (1.51–3.52)	
	60–74	0.88 (0.61–1.26)	0.89 (0.64–1.24)		0.85 (0.52–1.37)	0.86 (0.55–1.34)	
	75 or more	Reference	Reference		Reference	Reference	
Gender	Female	2.15 (1.77–2.62)	2.01 (1.68–2.4)	**<0.001**	2.24 (1.72–2.9)	2.01 (1.61–2.52)	**<0.001**
	Male	Reference	Reference		Reference	Reference	
Nationality	Local	0.54 (0.43–0.68)	0.57 (0.46–0.7)	**<0.001**	0.53 (0.38–0.72)	0.56 (0.42–0.75)	**<0.001**
	Expatriates	Reference	Reference		Reference	Reference	
History of COVID-19 before vaccination	No	Reference	Reference	**0.002**	Reference	Reference	0.565
	Yes	1.67 (1.21–2.3)	1.59 (1.2–2.1)		1.17 (0.69–1.98)	1.15 (0.72–1.81)	
History of chronic disease/s	No	Reference	Reference	0.333	Reference	Reference	**0.015**
	Yes	1.11 (0.9–1.37)	1.1 (0.91–1.34)		0.71 (0.53–0.93)	0.74 (0.58–0.94)	
Obesity (BMI 30 or more)	No	Reference	Reference	**0.005**	Reference	Reference	0.356
	Yes	1.44 (1.12–1.85)	1.39 (1.11–1.75)		1.18 (0.83–1.68)	1.15 (0.85–1.57)	

OR, Odds ratio; RR, Relative risk.

^*^Using Chi square test or Fisher exact test as appropriate. Statistically significant values *P* < 0.05.

In the context of the second dose, individuals below 60 exhibited a greater tendency to experience side effects than those aged 60 and above. When assessing the influence of gender and nationality, we found that females and expatriates demonstrated a markedly higher incidence of side effects compared to males and locals respectively. Intriguingly, individuals with a documented history of chronic diseases were less inclined to report side effects post the second dose (Table 2).

### 3.4 Predictors of side effects following BNT162b2 vaccination

Across both the first and second doses of the BNT162b2 vaccine, gender emerged as a consistent predictor of side effects. Females consistently exhibited a heightened likelihood of experiencing side effects. For the first dose, the association was significant with an adjusted odds ratio (AOR) of 2.19 (95%CI: 1.79–2.69, *p* < 0.001). This trend persisted for the second dose, where the AOR was 2.16 (95%CI: 1.64–2.83, *p* < 0.001) as shown in Table 3. Nationality also played a pivotal role in determining the likelihood of side effects. Interestingly, locals were less likely to develop side effects than expatriates. This was evident for both doses, with the first dose showing an AOR of 0.48 (95%CI: 0.38–0.61, *p* < 0.001) and the second dose yielding an AOR of 0.51 (95%CI: 0.37–0.72, *p* < 0.001). For the first dose, individuals with a history of COVID-19 infection prior to vaccination exhibited an increased risk of side effects, with an AOR of 1.86 (95%CI: 1.34–2.58, *p* < 0.001). Additionally, obesity (BMI ≥ 30) was associated with a marginally increased likelihood of side effects, with an AOR of 1.32 (95%CI: 1.01–1.73, *p* = 0.041) (Table 3).

**Table 3:** Predictors of developing one or more side effects following the first and second doses of BNT162b2 vaccine.

Characteristics		Any side effect							
		First dose				Second dose			
		Adjusted OR (95%CI)	*p*-value*	Adjusted RR (95%CI)	*p*-value^†^	Adjusted OR (95%CI)	*p*-value*	Adjusted RR (95%CI)	*p*-value^†^
Age	less than 30	0.87 (0.5–1.51)	0.612	0.88 (0.53–1.46)	0.615	1.03 (0.33–3.2)	0.954	1.02 (0.37–2.78)	0.976
	30–44	1.22 (0.83–1.81)	0.311	1.2 (0.85–1.69)	0.309	2.05 (1.23–3.4)	**0.006**	1.86 (1.19–2.91)	**0.006**
	45–59	1.02 (0.69–1.49)	0.924	1.03 (0.73–1.45)	0.872	2.47 (1.53–3.98)	**<0.001**	2.15 (1.41–3.27)	**<0.001**
	60–74	0.84 (0.58–1.21)	0.350	0.86 (0.62–1.2)	0.379	0.85 (0.52–1.39)	0.515	0.86 (0.55–1.35)	0.514
	75 or more	Reference		Reference		Reference		Reference	
Gender	Female	2.19 (1.79–2.69)	**<0.001**	2.02 (1.69–2.42)	**<0.001**	2.16 (1.64–2.83)	**<0.001**	1.9 (1.51–2.38)	**<0.001**
	Male	Reference		Reference		Reference		Reference	
Nationality	Local	0.48 (0.38–0.61)	**<0.001**	0.52 (0.42–0.64)	**<0.001**	0.51 (0.37–0.72)	**<0.001**	0.55 (0.42–0.74)	**<0.001**
	Expatriate	Reference		Reference		Reference		Reference	
History of COVID-19 before vaccination	No	Reference		Reference	**<0.001**	Reference		Reference	0.329
	Yes	1.86 (1.34–2.58)	**<0.001**	1.7 (1.29–2.24)		1.26 (0.73–2.17)	0.402	1.25 (0.8–1.95)	
History of chronic disease/s	No	Reference		Reference	0.344	Reference		Reference	0.504
	Yes	1.12 (0.89–1.41)	0.342	1.11 (0.9–1.36)		0.9 (0.66–1.21)	0.475	0.92 (0.72–1.18)	
Obesity (BMI 30 or more)	No	Reference		Reference	**0.044**	Reference		Reference	0.789
	Yes	1.32 (1.01–1.73)	**0.041**	1.27 (1.01–1.61)		1.09 (0.74–1.6)	0.656	1.04 (0.76–1.43)	

OR, Odds ratio; RR, Relative risk.

^*^Using multivariable logistic regression.

^†^Using multiple log-binomial regression.Statistically significant values *P*<0.05.

## 4 Discussion

Vaccine reactogenicity encompasses diverse local and systemic expressions arising from the inflammatory reaction triggered by vaccination. The extent of reactogenicity is influenced by factors such as host characteristics (age, gender, etc.), vaccine type, composition, route of administration, and several other variables (28). This study is the first to focus on post-vaccination side effects linked to the most commonly used COVID-19 vaccine, BNT162b2, in Qatar. The findings presented in this study offer insights into the sociodemographic characteristics, distribution, determinants, and predictors of side effects following vaccination in a diverse population of Qatar.

The age distribution of the participants mirrors the progression of COVID-19 vaccination uptake in Qatar (29). During the initial phases of vaccination, emphasis was placed on prioritizing individuals at higher risk, particularly older adults. The gender distribution, comprising about 29% females, reflects the actual proportion of females in the population of Qatar, enhancing the generalizability of the results (30).

Our study indicated significantly lower percentage (less than 12%) of participants experiencing at least one side effect within 3–5 days after COVID-19 vaccination compared to other international and regional studies (7, 31–35), which reported higher but heterogenous percentages ranging from 25 to 100%. This may be attributed to several factors. Firstly, the study was conducted within a narrow timeframe (3–5 days after vaccination) which might have hindered the ability to catch the side effects that could have developed after day 5 of the vaccination. Secondly, research has shown that Qatar has a relatively low vaccine hesitancy, reported at 20% in one study (36). Additionally, a literature review in the Gulf region showed that Qatar has one of the lowest vaccine hesitancy rates in the region (37). Furthermore, the high transmission rate of COVID-19 infection reported in Qatar, and the heightened risk of individuals contracting COVID-19, could have influenced the population’s inclination to actively participate in vaccination, viewing it as a crucial intervention to combat the virus. This situation could introduce a bias in reporting vaccine side effects, as individuals might be more pushed to perceive the intervention positively and ignore its adverse reactions.

Injection site pain emerged as the most commonly reported local symptom after the first and second vaccine doses in our study, followed by injection site swelling at notably lower frequencies, aligning with findings from other studies (7, 31, 32). Regarding systemic adverse effects, our analysis revealed that headache, fever, and fatigue were among the most prevalent systemic symptoms after both vaccine doses, which is consistent with other studies and reports (7, 31–35).

The results of our study revealed a higher likelihood of experiencing a side effect after the second dose compared to the first one, which is consistent with other studies and systematic reviews (7, 31, 35, 38, 39). On the other hand, other types of vaccines (like Sputnik V, Oxford-AstraZeneca [ChAdOx1-S/nCoV-19], or Sinopharm [BBIBP-CorV]) showed opposite patterns (40). Upon further analysis, it was determined that local side effects after the first dose were 1.6 times more likely to occur than after the second dose. On the other hand, systemic side effects were approximately three times more prevalent after the second dose than after the first one. These findings remain consistent even after adjusting for individuals’ COVID-19 history. This is aligned with other studies (38, 39). Similarly, a study in UAE showed that among the mRNA vaccine recipients, the number of side effects reported after the second dose was 2.6 times higher than after the first dose of the vaccine (33). A study conducted at Sidra Hospital in Qatar revealed a heightened amplitude of immune responses after the second dose, and the identification of an inflammatory component corresponds to the reported increase in the occurrence of side effects or discomfort following the administration of the second dose of the COVID-19 mRNA vaccine (41).

Our findings indicate that individuals with a history of prior COVID-19 infection before the first vaccine dose are nearly twice as likely to experience side effects compared to those without such a history which is consistent with other studies (39, 42–44). Those with a prior history of COVID-19 infection are almost three times more likely to experience systemic side effects compared to those without such a history. In contrast, this difference is not clearly evident for local side effects. These observations align with the findings of Krammer et al. (42) and Chaudhary et al. (44). This can suggest a potential interplay between pre-existing immunity and vaccine-induced responses. The increased side effects observed in this subgroup may be ascribed to an augmented immune response activated by natural infection and vaccination. This underscores the importance of developing customized vaccination strategies for individuals with a history of prior infection, as their immune system is already primed to respond to the virus (45). Moreover, vaccinating individuals with documented evidence of a prior infection appears to elicit a booster response, resulting in IgG titers approximately one order of magnitude higher than those observed in individuals without previous exposure (46, 47). These findings align with emerging real-world evidence indicating that individuals with prior SARS-CoV-2 infection exhibit antibody responses to the first vaccine dose comparable to or surpass the antibody titers observed in individuals without previous infection after receiving the second dose (48).

The age-related variations in side effects following the second dose indicate a nuanced response to the second dose, particularly among individuals below the age of 60 who exhibit a higher propensity for side effects. Interestingly, we did not observe significant differences between age categories after the first dose. Aligning with our results, findings from other studies also suggested that the younger age group is more susceptible to developing side effects for both doses (7, 49). Additionally, a systematic review demonstrated that individuals aged ≤ 55 years faced a significantly higher risk of side effects than those aged ≥ 56 years, with a pooled relative risk of 1.25 (95% CI 1.15–1.35, *p* < 0.001) (34). As individuals age, the function of the immune system undergoes a decline, a phenomenon known as immunosenescence (50). This age-related change involves the gradual loss of naive cells, an increase in memory cell numbers, and a decrease in the diversity of T and B cell repertoires (51). Consequently, older adults exhibit reduced protection against infectious diseases and diminished vaccine responses. In response to immunization, both inflammatory reactions and protective immune responses in the older adult population are slower, weaker, and more transient than in younger, healthy adults (52). Despite these observations, the molecular mechanisms underlying age-related hyporesponsiveness to vaccination remain unclear.

Our study identified gender and nationality as significant predictors of the likelihood of experiencing side effects following the BNT162b2 vaccine after both doses. Notably, females demonstrated a heightened probability of encountering side effects with nearly double the likelihood compared to males for both local and systemic side effects. This aligns with findings from other studies (53, 54). Clinical data highlights the impact of gender on the frequency and severity of vaccination-related adverse reactions, including fever, pain, and inflammation (55). The observed sex differences in humoral immune response across various vaccines underline the need for inclusive recruitment in vaccine trials to assess potential clinical implications of gender disparities (56). Additionally, research done in 2019 indicated that women developed stronger cytokine and antibody responses than men after receiving the flu vaccine (57). In contrast to our results, a study conducted in Saudi Arabia reported that males experienced more adverse effects than females (58), possibly influenced by reporting behaviors. Numerous psychosocial and biological factors contribute to gender disparities in the rates of vaccine side effects. Pain, as one of the most common side effects, is influenced by gender differences in pain sensitivity, which can be attributed to endogenous opioids, genetics, and the modulatory influence of sex hormones. Stereotypical sex roles and psychosocial processes such as stress exposure and pain coping mechanisms also play a role in differences in pain threshold between genders (59, 60). The lower COVID-19 case-fatality rates in women compared to men (61) possibly due to an enhanced immune response in women, which could be part of the explanation of the higher frequency of side effects in females due a stronger immediate response (62). Women typically exhibit higher expression of type IFN I, innate immune responses, and T cell-associated genes (62). Additionally, sex hormones play a major role, particularly testosterone, which can depress the immune response, potentially explaining the higher frequency of adverse events in females following vaccination (63). Genetic factors, including those related to the ACE2 and Ang-II receptor type 2 genes located on the X chromosome, may also interact with sex hormones to increase immune response in females and contribute to vaccine-associated adverse events (64). Additionally, factors such as healthcare-seeking behavior, reporting bias, and societal roles may contribute to differences in reported vaccine side effects between females and males.

Our study found that a history of chronic diseases did not emerge as a significant predictor of side effects, consistent with findings in other studies (54). The association between obesity (BMI ≥ 30) and increased adverse outcomes post-vaccination is noteworthy. Our findings indicate a significant association between obesity and the likelihood of side effects after BNT162b2 vaccines, contradicting conclusions drawn in other studies (65, 66). However, this supports findings from other studies suggesting a potential association (67, 68). The literature emphasizes the current lack of conclusive evidence regarding vaccine safety in the obese population (69). Further research is necessary to delve into the mechanisms underlying this potential association.

### 4.1 Strengths and limitations

This study is the first to assess COVID-19 vaccination side effects in Qatar, bridging a crucial gap in local data on COVID-19 vaccines. The meticulously crafted methodology contributes essential information for informed public health decision-making in Qatar for future events. However, there are some limitations to consider. The study primarily focused on short-term side effects within the initial 3–5 days post-administration of the BNT162b2 COVID-19 vaccine, potentially overlooking delayed reactions. Some other studies have encompassed extended observation periods within the short-term side effects category, complicating comparability. However, evidence suggests that the majority of short-term side effects following vaccination manifest within the first 3 days post-administration. It’s essential to note that the data form used for collection lacks information on the severity of each side effect, preventing the derivation of conclusions about the intensity of patients’ experiences and related factors. The retrospective design of the study creates challenges in conducting timely follow-ups on patients, thereby impeding exploration of side effects details, including duration and resolution. Additionally, the absence of a control group poses difficulties in establishing a definitive causative link between the observed side effects and the administered vaccine. The potential for misclassification bias exists, as individuals with chronic diseases who did not access public health care services or exclusively used private facilities were labeled as having no chronic disease due to the absence of recorded encounters. Furthermore, the restricted access to complete medical records posed challenges in assessing treatment variations, medications, and the duration of participants’ interactions with the medical system. Another limitation stems from the lack of data on the severity of COVID-19 infections in individuals previously diagnosed, restricting the inclusion of a crucial variable in the analysis.

## 5 Conclusion

In summary, this study offers a detailed analysis of short-term side effects following Pfizer-BioNTech (BNT162b2) COVID-19 vaccination in Qatar, revealing a relatively low incidence of side effects. Notably, gender, nationality, age, prior COVID-19 infection, and obesity emerged as significant predictors of side effects. Additionally, there is an observed opposite pattern in the proportions of individuals experiencing side effects after the first dose compared to the second dose, in relation to systemic and local side effects. Post-first-dose local side effects were more prevalent compared to the second dose, while the opposite relationship was observed for systemic effects. The study investigated the determinants and predictors of vaccine side effects, highlighting their significant impact on side effect occurrence. It suggests that future research should prioritize these factors to develop evidence-based personalized vaccination strategies. For instance, customizing doses or frequencies for certain groups, like females or individuals with prior infections, could minimize side effects while maintaining effectiveness. Moreover, identifying individuals needing close side effect monitoring could optimize resource allocation and enable preventive measures, ultimately reducing side effect occurrence. This approach could also help mitigate vaccine hesitancy and non-acceptance.

## Data availability statement

The raw data supporting the conclusions of this article will be made available upon reasonable request by the corresponding author.

## Ethics statement

The studies involving humans were approved by Ethical approval was obtained from the Primary Health Care Corporation research committee with protocol ID (PHCC/DCR/2022/04/024). The studies were conducted in accordance with the local legislation and institutional requirements. Written informed consent for participation was not required from the participants or the participants’ legal guardians/next of kin in accordance with the national legislation and institutional requirements.

## Author contributions

SA: Conceptualization, Data curation, Formal analysis, Methodology, Writing – original draft, Writing – review & editing. MA: Conceptualization, Formal analysis, Methodology, Writing – original draft, Writing – review & editing. MA-Z: Conceptualization, Methodology, Supervision, Writing – review & editing. MM: Conceptualization, Methodology, Writing – review & editing. JA: Data curation, Formal analysis, Writing – review & editing. AA-N: Conceptualization, Writing – review & editing, MA-K: Conceptualization, Methodology, Supervision, Writing – review & editing.
