# Supplementary material for: Short-term side effects of BNT162b2 vaccine in primary care settings in Qatar: a retrospective study
Source: Front Public Health. 2024 Apr 10;12:1384327. doi: 10.3389/fpubh.2024.1384327 (PMC11039914; doi:10.3389/fpubh.2024.1384327)
Supplement: Supplementary file 1 [file Table_1.DOCX]

Supplementary Table 1: Comparison of the proportions of different side effects experienced by participants between the first and second BNT162b2 vaccine doses.

| **Side effects** | **First dose**  **No (%)** | **Second dose**  **No (%)** | **P-value*** | **Odds ratio**  **(second to first dose)** | **Relative risk**  **(second to first dose)** |
| --- | --- | --- | --- | --- | --- |
| **Local side effects** | 303 (5.5) | 88 (3.9) | **0.002** | 0.69 (0.54-0.88) | 0.7 (0.56-0.88) |
| **Injection site pain** | 283 (5.2) | 80 (3.5) | **0.002** | 0.67 (0.52-0.86) | 0.68 (0.53-0.87) |
| **Injection site redness** | 13 (0.2) | 4 (0.2) | 0.601 | 0.74 (0.24-2.28) | 0.74 (0.24-2.27) |
| **Injection site swelling** | 22 (0.4) | 7 (0.3) | 0.540 | 0.77 (0.33-1.8) | 0.77 (0.33-1.79) |
| **Localized Swollen lymph nodes** | 6 (0.1) | 4 (0.2) | 0.457 | 1.61 (0.45-5.71) | 1.61 (0.45-5.69) |
| **Systemic side effects** | 193 (3.5) | 208 (9.1) | **<0.001** | 2.76 (2.26-3.38) | 2.6 (2.15-3.14) |
| **Fatigue and tiredness** | 48 (0.9) | 47 (2.1) | **<0.001** | 2.39 (1.59-3.59) | 2.36 (1.58-3.52) |
| **Fever** | 57 (1.0) | 107 (4.7) | **<0.001** | 4.7 (3.4-6.51) | 4.53 (3.3-6.22) |
| **Headache** | 71 (1.3) | 70 (3.1) | **<0.001** | 2.42 (1.73-3.38) | 2.38 (1.72-3.3) |
| **Myalgia** | 25 (0.5) | 42 (1.8) | **<0.001** | 4.11 (2.5-6.76) | 4.05 (2.48-6.63) |
| **Dizziness** | 17 (0.3) | 10 (0.4) | 0.376 | 1.42 (0.65-3.11) | 1.42 (0.65-3.09) |
| **Chills** | 13 (0.2) | 17 (0.7) | **0.001** | 3.17 (1.54-6.54) | 3.16 (1.54-6.49) |
| **Nausea** | 12 (0.2) | 12 (0.5) | **0.026** | 2.42 (1.09-5.4) | 2.41 (1.09-5.36) |
| **Vomiting** | 4 (0.1) | 1 (0) | >0.999 | 0.6 (0.07-5.4) | 0.6 (0.07-5.39) |
| **Abdominal pain** | 6 (0.1) | 6 (0.3) | 0.122 | 2.42 (0.78-7.5) | 2.41 (0.78-7.47) |
| **Diarrhea** | 0 (0) | 0 (0) | ---- | ---- | ---- |
| **Arthralgia** | 3 (0.1) | 13 (0.6) | **<0.001** | 10.51 (2.99-36.92) | 10.46 (2.98-36.65) |
| **Both local and systemic side effects** | 442 (8.1) | 266 (11.7) | **<0.001** | 1.51 (1.29-1.78) | 1.45 (1.26-1.68) |

* Using Chi square test or Fisher exact test as appropriate
